# Low Pathogenicity H7N3 Avian Influenza Viruses Have Higher Within-Host Genetic Diversity Than a Closely Related High Pathogenicity H7N3 Virus in Infected Turkeys and Chickens

**DOI:** 10.3390/v14030554

**Published:** 2022-03-08

**Authors:** Christina M. Leyson, Miriã F. Criado, Sungsu Youk, Mary J. Pantin-Jackwood

**Affiliations:** Exotic and Emerging Avian Viral Diseases Research Unit, Southeast Poultry Research Laboratory, United States National Poultry Research Center, Agricultural Research Service, United States Department of Agriculture, Athens, GA 30605, USA; christina.leyson@usda.gov (C.M.L.); mfc0034@auburn.edu (M.F.C.); sungsu.youk@usda.gov (S.Y.)

**Keywords:** low pathogenicity avian influenza, high pathogenicity avian influenza, within-host diversity, virus evolution, high throughput sequencing, chickens, turkeys

## Abstract

Within-host viral diversity offers a view into the early stages of viral evolution occurring after a virus infects a host. In recent years, advances in deep sequencing have allowed for routine identification of low-frequency variants, which are important sources of viral genetic diversity and can potentially emerge as a major virus population under certain conditions. We examined within-host viral diversity in turkeys and chickens experimentally infected with closely related H7N3 avian influenza viruses (AIVs), specifically one high pathogenicity AIV (HPAIV) and two low pathogenicity AIV (LPAIVs) with different neuraminidase protein stalk lengths. Consistent with the high mutation rates of AIVs, an abundance of intra-host single nucleotide variants (iSNVs) at low frequencies of 2–10% was observed in all samples collected. Furthermore, a small number of common iSNVs were observed between turkeys and chickens, and between directly inoculated and contact-exposed birds. Notably, the LPAIVs have significantly higher iSNV diversities and frequencies of nonsynonymous changes than the HPAIV in both turkeys and chickens. These findings highlight the dynamics of AIV populations within hosts and the potential impact of genetic changes, including mutations in the hemagglutinin gene that confers the high pathogenicity pathotype, on AIV virus populations and evolution.

## 1. Introduction

RNA viruses are known to have high mutation rates and short generation times. Thus, at any given time, viruses can be considered as populations of genetic variants [1,2]. Under the appropriate conditions, variants that are present at a low frequency can replicate exponentially and quickly become predominant in the population. The existence of such variants in a population allows for rapid response to prevailing circumstances and thereby can potentially lead to the emergence of new viral strains [3,4].

Viral genetic diversity can be examined at various timescales. Phylodynamic analyses often examine sequences across months or years, giving an epidemiologic perspective to viral evolution. On a shorter timeframe, within-host virus diversity can provide insight into the early steps of viral evolution [4]. Because within-host virus diversity can be considered as the foundation from which the genetic diversity originates across time and space, investigating at the within-host level enables us to explore virus evolution in its earliest stages, soon after infecting a host.

The sensitivity at which we can detect minor genetic variations has increased along with the development of accessible, cost-effective methods for high throughput deep sequencing [4,5]. Moreover, deep sequencing has allowed investigators to identify small low-frequency variations across a substantial number of samples, particularly single nucleotide variations (SNVs). Within-host SNVs, also known as intra-host SNVs (iSNVs), have been examined for a number of virus species, including influenza viruses [6,7,8,9,10,11,12], Newcastle disease virus [13], human immunodeficiency virus (HIV) [14,15,16], hepatitis C virus (HCV) [17,18], foot-and-mouth disease virus (FMDV) [19], severe acute respiratory disease coronavirus 2 (SARS-CoV-2) [20], and herpes simplex virus (HSV-1) [21]. Examination of within-host diversity has provided insights on the emergence of phenotypic traits such as antiviral drug resistance [14,17,18,22], the transmission of viruses between hosts [8,9,19,20], and viral spread to multiple tissues in a single host [23].

Influenza A viruses, which are RNA viruses with segmented genomes, have a rich genetic diversity due not only to their high mutation rate but also to their ability to reassort genome segments [24]. Wild waterfowl are reservoirs of avian influenza viruses (AIVs), and nearly all subtypes exist in waterfowl species [25]. Host susceptibility to AIVs can vary widely depending on the AIV strain and host factors including species, age, previous AIV exposure, physiological stress, and immune suppression. AIVs periodically spread from wild waterfowl to poultry, causing outbreaks that can significantly impact poultry production and result in large economic losses. AIVs can be classified into low pathogenicity (LP) or high pathogenicity (HP) AIVs according to the severity of disease observed in gallinaceous species such as chickens and turkeys [25]. The presence of a multibasic cleavage site (MBCS) in the hemagglutinin (HA) gene is the key mutation observed in HPAIVs that enables the virus to replicate and spread systemically [26]. Currently, only H5 and H7 AIV subtypes are known to gain a MBCS and thereby evolve to the HPAIV form. Indeed, major outbreaks in poultry have been caused by H5 and H7 HPAIVs [25].

In the United States (US), H7 subtype AIVs have caused several outbreaks in poultry with some leading to the emergence of HPAIVs [27,28,29,30,31]. Many of these outbreaks have affected turkeys. Moreover, turkeys have been experimentally shown to be susceptible to influenza viruses from other species including waterfowl, swine, and human-origin influenza viruses [32,33]. Thus, turkeys can play an important role as an intermediate host between wild birds and other domestic avian species and can be a key driver of AIV outbreaks [34]. It is therefore important to understand the changes H7 subtype AIVs undergo when replicating in turkeys and how this affects virus fitness, evolution, and the emergence of viruses capable of causing outbreaks in poultry.

The main objective of this study was to investigate within-host virus diversity of three closely related H7N3 AIVs in turkeys and chickens. In a previous study, we examined the pathogenicity in these two poultry species of three H7N3 AIVs from the 2020 outbreak that occurred in commercial turkeys in North and South Carolina, US [30,35]. Phylogenetic analyses of the viruses involved in the outbreak showed that a single introduction of a wild bird AIV had occurred, and thus all viruses from this H7N3 outbreak have high genetic similarity to each other [30]. Turkeys and chickens were inoculated with either a HPAIV or one of two different LPAIVs with different neuraminidase (NA) stalk lengths. Clear differences were observed between these two species, with turkeys requiring a lower virus dose to become infected, and infected turkeys efficiently transmitting the virus to contacts [35]. For this study, we conducted deep sequencing of virus genomes from oropharyngeal (OP) swabs and tissue samples collected from the experimentally infected birds to examine the diversity of viral genomes and to provide insights into AIV within-host populations. Specifically, we compared the within-host virus diversity between genomes from the three closely related H7N3 viruses by examining how the following host and virus factors impacted single nucleotide variants (iSNV) diversity: host species (turkeys versus chickens), inocula dose, route of exposure (inoculated versus contact-exposure), time after exposure, sample type (swabs versus tissues), and virus pathotype (HPAIV or LPAIV).

## 2. Materials and Methods

### 2.1. Experimental Samples

Samples collected from turkeys and chickens inoculated with A/turkey/South Carolina/20-008394-001/2020 (H7N3) (LPAIV-1), A/turkey/North Carolina/20-008425-001/2020 (H7N3) (LPAIV-2), or A/turkey/South Carolina/20-010561-006/2020 (H7N3) (HPAIV) were analyzed in this study. These H7N3 viruses were isolated during the 2020 US H7N3 AIV outbreak in turkeys [30]. In the pathogenesis study [35], birds were intrachoanally inoculated with an assigned virus inocula of 2, 3, 4, 5, or 6 log_10_ EID_50_ (50% egg infectious dose) and were monitored for clinical signs and mortality. Naïve contact birds were co-housed with directly inoculated birds 24 h post-inoculation to determine virus transmissibility. Oropharyngeal (OP) and cloacal swabs were collected from 0.5 to 14 days post-inoculation (dpi). Necropsy was performed on three birds per group, and tissues (brain, heart, lung, muscle, and spleen) were collected at 2 days dpi. Quantitative real-time RT-PCR (qRT-PCR) was subsequently performed to measure viral RNA in swab and tissue samples [36].

### 2.2. Whole-Genome Sequencing of Avian Influenza Viruses

Whole-genome sequencing was carried out as previously described [10]. Amplification of full-genome segments were conducted with samples that had a Ct value of less than or equal to 28, which was shown to result in whole genome sequencing success [37]. Most of the cloacal samples, swabs from contact-exposed chickens, and tissues from LPAIV-inoculated birds had low viral RNA titers and thus were not used for whole genome sequencing. For each sample, two hundred fifty microliters of chorioallantoic fluid used as virus inoculum, OP swab media, or 10% (*w/v*) tissue homogenates were subjected to total RNA extraction using Trizol LS (Thermo Fisher Scientific, MA, USA) and RNA Clean and Concentrator-96 (Zymo Research, CA, USA). The total RNA samples were then used as a template for RT-PCR using Superscript IV One-step RT-PCR kit (Thermo Fisher Scientific) and Opti F1, Opti F2, and Opti R primers at a 35:65:100 molar ratio. Primer sequences are available at [10]. The RT-PCR products were purified using Agencourt AMPure XP beads (Beckman Coulter, CA, USA) at a 1.8:1 (*v/v*) bead: RT-PCR product ratio. The purified cDNAs were then fragmented and barcoded using the Nextera XT DNA library prep kit (Illumina, CA, USA) and sequenced using a 500-cycle Miseq Reagent kit v2 on an Illumina Miseq instrument (2 × 250 bp, Illumina).

### 2.3. Viral Genome Assembly

Whole genomes were assembled using a previously described workflow [38] on a local Galaxy server [39]. In this workflow, paired reads were merged using PEAR [40] and reference mapping to the consensus sequences of the inoculum was done using BWA-MEM [41,42].

### 2.4. Determination of iSNVs

Sequence variant frequencies were then determined using Lofreq [43] and the consensus sequences obtained from whole-genome sequencing of virus inocula as reference sequences. Lofreq is a highly sensitive variant caller that takes into account base read quality scores [43] and has been used in previous studies with the influenza virus [10,44,45]. For all measures of within-host virus diversity, only single nucleotide intra-host variations (iSNVs) at 2% or higher frequencies were included in the following analyses to avoid false positives. This cutoff was identified in previous studies [44,46,47] and reviewed in [4]. The Lofreq default setting of 10 for the minimum read depth was used.

### 2.5. Calculations for Mean iSNV Frequency and Proportion

Given that *p_k_* is the iSNV frequency of the *k*th iSNV and *n* is the total number of iSNVs found in each sample, the mean iSNV frequency per sample is calculated as $\frac{1}{n}\sum_{k=1}^{n}p_{k}$. The mean nonsynonymous iSNV frequency is calculated in a similar manner, but only nonsynonymous iSNV changes are considered, i.e., $\frac{1}{m}\sum_{k=1}^{m}p_{k}$ where *p_k_* is the frequency of the nonsynonymous iSNV and *m* is the total number of nonsynonymous iSNVs in each sample. Lastly, the proportion of nonsynonymous iSNVs *(X*) in a sample is calculated as $X=\frac{m}{n}$ where *m* is the total number of nonsynonymous iSNVs and *n* is the total number of iSNVs in each sample.

### 2.6. Diversity Measures

Two measures of within-host virus diversity, Shannon entropy [44,46] and nucleotide diversity [47], were calculated as previously described. Given than *p_i_* is the iSNV frequency of the *i*th allele at position *l*, Shannon entropy was calculated as $-\sum_{l=1}^{L}H_{l}/L\;\mathrm{where}\;H_{l}=\sum_{i=1}^{4}p_{i}(\log\;p_{i})\;$and nucleotide diversity is calculated as $\pi =\sum_{l=1}^{L}D_{l}/L\;\mathrm{where}\;D_{l}=1-\sum_{i=1}^{4}p_{i}^{2}$. The concatenated genome length *L* for each of the viruses used are 13,608 bp for HPAIV, 13,581 bp for LPAIV-1, and 13,515 bp for LPAIV-2. Custom Python scripts were written to enumerate SNVs and calculate mean SNV frequency, Shannon entropy, and nucleotide diversity and are available at this website (https://github.com/cmleyson/H7N3_wgs, accessed on 16 December 2021). The Python version 3.8.5 and Conda version 4.10.1 were used (https://anaconda.com, accessed on 3 September 2021).

### 2.7. Statistics

All statistical tests were performed using the SciPy library version 1.7.1. Kruskal-Wallis and Dunn’s post hoc test were used in statistical tests for iSNV frequencies, proportions, and diversity comparing the three H7N3 viruses. Mann-Whitney test was used in statistical tests for iSNV frequencies, proportions, and diversity comparing between swab and tissue samples.

## 3. Results

The 2020 H7N3 US AIV outbreak in commercial turkeys was most likely initiated by a LPAIV spill over event from wild birds into a turkey flock. The spread and replication of the LPAIV among turkeys eventually resulted in the emergence of a HPAIV by the acquisition of basic amino acids at the hemagglutinin (HA) cleavage site. The H7N3 LPAIV-1, LPAIV-2, and HPAIV used in this study, as well as all viruses sequenced from the outbreak, were shown to be highly related to each other, with over 99 percent sequence identity across all segments [30,35]. LPAIV-1 and HPAIV belong to the major group of viruses identified from the outbreak called Cluster A, while LPAIV-2 belongs to a smaller group of viruses called Cluster B, in which the neuraminidase (NA) segment had a 66-nucleotide deletion in the stalk region of the NA gene [30].

In our prior pathogenesis study in turkeys and chickens, the mean bird infectious dose (BID_50_) of these H7N3 viruses was found to be markedly lower for all three viruses in turkeys (<2–2.5 log_10_ EID_50_) compared to chickens (3.7–5.7 log_10_ EID_50_). Moreover, virus-inoculated turkeys transmitted the viruses to all contacts regardless of the virus doses tested, whereas transmission was only observed in chickens inoculated with either of the two LPAIVs at the highest virus doses tested. These results demonstrate that turkeys are more susceptible to the 2020 H7N3 viruses compared to chickens [35].

### 3.1. Whole Genome Sequencing from Birds Experimentally Infected with H7N3 AIVs

To examine within-host variations of the H7N3 viruses, we performed deep sequencing of AIVs in oropharyngeal (OP) swabs and tissues from experimentally infected turkeys and chickens after inoculation or contact exposure with one of the three 2020 H7N3 viruses. A total of 180 samples were successfully sequenced from OP swab and tissue samples (Table 1). Only a few cloacal (CL) swab samples had sufficient viral RNA to successfully amplify and sequence the genome, and thus the CL swabs were not included in this study. Likewise, sequencing from contact-exposed birds was only successful from turkey samples due to low viral titers in contact-exposed chickens. Genome sequencing from infected tissues was also available only for HPAIV-infected birds because the two LPAIVs replicated poorly or did not replicate in organs as expected for LPAIVs. For each sample, the whole genome sequence was then assembled, and intra-host nucleotide variants (iSNV) identified.

### 3.2. Most iSNVs Were Nonsynonymous Mutations at Low-Frequency

**Figure 1:**
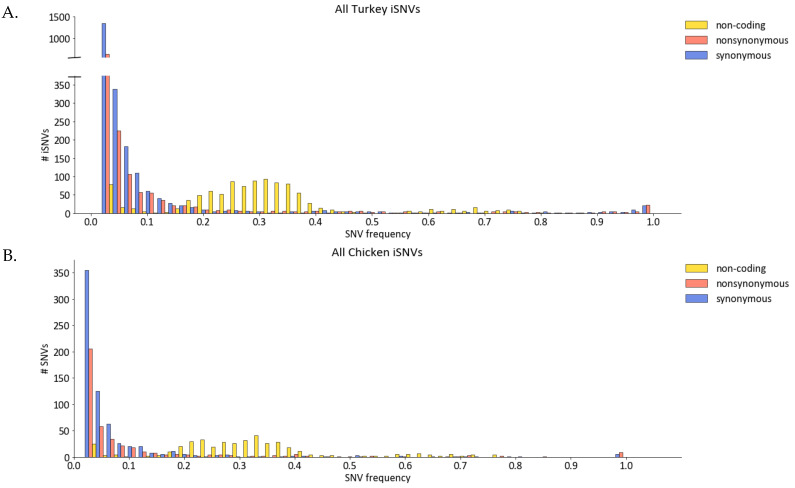
Histogram of iSNVs observed in samples from turkeys (**A**) and chickens (**B**) experimentally infected with the H7N3 AIVs. Each iSNV was classified as a nonsynonymous mutation if it resulted in an amino acid change in at least one viral protein.

A total of 3723 unique iSNVs were identified in the samples considered in this study, of which 2368 (63.6%) were nonsynonymous. The iSNVs in all virus groups were distributed across the entire length of the genome with some iSNVs found in multiple samples (Supplementary Figure S1). Most of iSNVs observed had frequencies below 50% (Figure 1 and Supplementary Figures S1 and S2), with an overall mean frequency of 14%. Indeed, the mean iSNV frequency for each segment and for each sample were generally below 50%, though there was a small number of samples where the mean iSNV frequencies in some segments were above 50% (Figure 1 and Supplementary Figures S1 and S3). However, comparison of iSNVs frequencies among segments was not significantly different (*p* > 0.05, Kruskal–Wallis test). Notably, there was a preponderance of noncoding iSNVs with 20–40% frequency at the beginning of each segment, many of which correspond to the position 4 that are known to have an A or G [48] (Figure 1 and Supplementary Figure S1).

### 3.3. Only a Few iSNVs Are Shared among Different Host Species and Contact-Exposed Groups

**Figure 2:**
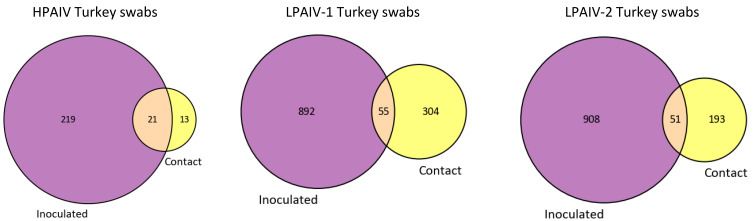
Unique iSNVs from viral genomes sequenced in OP swabs sampled from directly inoculated and contact turkeys. The iSNVs were enumerated and pooled for groups inoculated or contact-exposed with the same virus. The sets of iSNVs were then compared between samples from directly inoculated and contact turkeys.

Unique iSNVs from the virus genomes sequenced from samples in each species were identified to determine the richness of iSNV diversity. We observed that there was only a small proportion of iSNVs shared between turkeys and chickens for each group of samples sequenced (Supplementary Figure S3). Likewise, we also found that there were only small overlaps in the unique iSNVs shared between directly inoculated and contact turkeys, regardless of the virus (Figure 2). Because transmission between chickens was inefficient for the three H7N3 viruses used in the bird study, the analyses of iSNVs transmitted to contacts were not performed for chicken samples.

Considering all iSNVs from each of the three viruses used, there were five changes with ≥50% frequency that were found in at least one turkey and one chicken sample (Table 2). Four out of five of these changes were nonsynonymous. Furthermore, two of these five iSNVs, namely the nonsynonymous changes NS-G485A and NS-T294C, were found in virus sequences from both directly inoculated and contact turkeys at the 3 or 5 log_10_ EID_50_ dose of LPAIV-2 or HPAIV, respectively. To our knowledge, these two changes are not associated with a known phenotype. Further studies will be needed to determine whether these changes are potentially related to virus adaptation to gallinaceous species.

### 3.4. Comparison of iSNV Diversity with Respect to Host Species, Dose, Route of Exposure, and Time after Infection

It has been previously shown that within-host diversity of AIVs can vary depending on the host, species, route of exposure, and virus replication site [10,11]. Within-host diversities were thus compared for dose (2, 3, 4, 5, or 6 log_10_ EID_50_), route of exposure (inoculated or contact-exposed), and time after exposure for each virus (LPAIV-1, LPAIV-2, or HPAIV), but no significant differences were found. Specifically, we did not observe any significant differences in measures of iSNV diversity between directly inoculated and contact-exposed turkey groups (*p* > 0.05 for mean iSNV count, Shannon entropy, or nucleotide diversity). Nor did we observe any correlation between any measure of iSNV diversity and dose (−0.30 to 0.19, *p* > 0.05, Pearson correlation) or day of sample collection (−0.35 to 0.42, *p* > 0.05, Pearson correlation).

### 3.5. Virus Pathogenicity Type Can Impact Nonsynonymous Changes and iSNV Diversity in Virus Genomes from Directly Inoculated Birds

#### 3.5.1. Frequencies and Proportion of Nonsynonymous Changes Are Higher in LPAIVs than HPAIVs in Swabs from Directly Inoculated Birds

**Figure 3:**
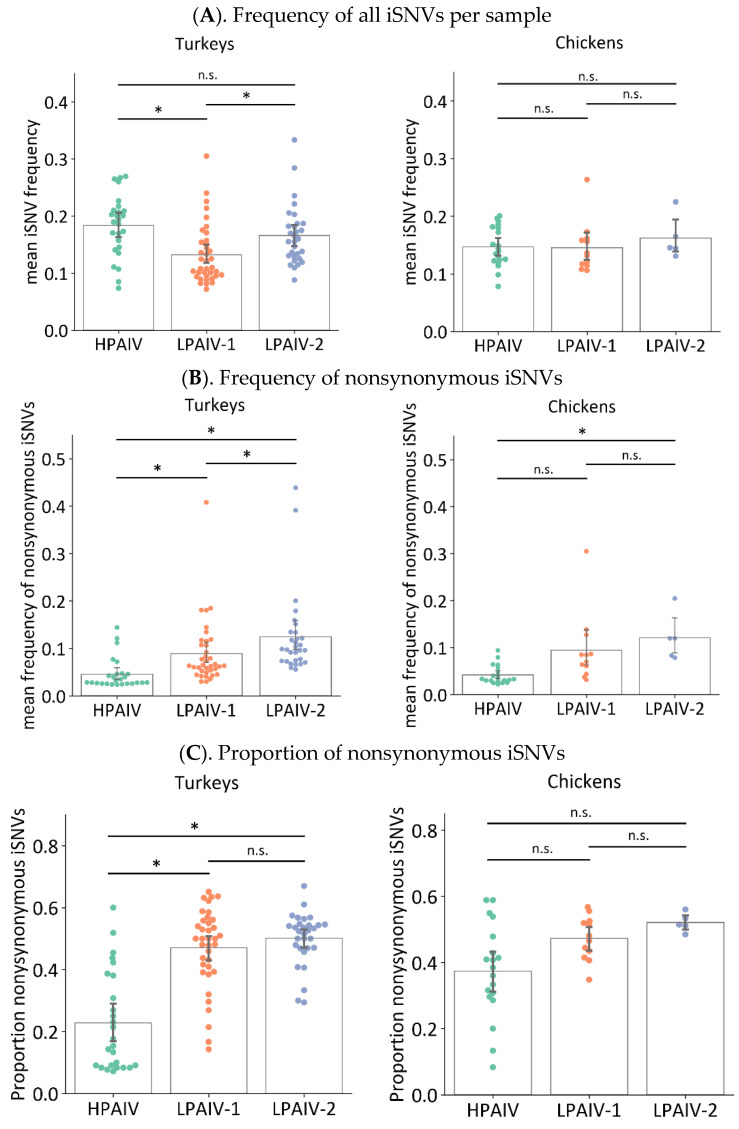
The iSNVs found in OP swabs from inoculated turkeys and chickens were classified according to whether the iSNV resulted in a change in amino acid (nonsynonymous) or not (synonymous). The overall mean iSNV frequency, regardless of whether it is nonsynonymous, was then calculated for each swab sample and compared among the three virus groups (**A**). To determine whether iSNVs from different virus groups resulted in different levels of nonsynonymous changes, the mean frequency (**B**) and proportion (**C**) of nonsynonymous changes were calculated. Asterisks (*) indicate *p* < 0.05 for indicated groups using Kruskal–Wallis and Dunn’s post hoc test for multiple comparisons. n.s. = not significant with *p* ≥ 0.05.

To determine how much change occurred during within-host virus replication, the mean iSNV frequency per sample was calculated in virus genomes sequenced in samples from directly inoculated birds. The mean iSNV frequency per sample is the average frequencies of all iSNVs that were detected in a sample. To calculate the mean iSNV frequency, the frequencies of iSNVs in a given category, i.e., all iSNVs or nonsynonymous iSNVs only, were added and then divided by the total number of iSNVs in the said category. A high mean iSNV frequency indicates that, on average, the iSNVs in a sample is farther in the sequence relative to that of the inocula [10]. When both nonsynonymous and synonymous changes were considered, the mean iSNV frequency in OP swabs from directly inoculated turkeys infected with LPAIV-2 or HPAIV was significantly higher compared to than that of LPAIV-1 (Figure 3A, Table 3). No differences were observed in virus genomes in samples from turkeys infected with HPAIV or LPAIV-2, as well as for the OP swab samples from chickens infected with any virus. In contrast, when only nonsynonymous changes are considered, samples from HPAIV-infected turkeys had significantly lower mean nonsynonymous iSNV frequency than that from LPAIV-1- or LPAIV-2-infected turkeys (Figure 3B, Table 3). Moreover, LPAIV-2 samples in turkeys had higher mean nonsynonymous iSNV frequency than LPAIV-1 samples. For chickens, the HPAIV genomes had lower mean frequency of nonsynonymous iSNVs compared to LPAIV-2 but not LPAIV-1 (Figure 3B, Table 3).

The proportion of nonsynonymous iSNVs relative to all iSNVs for each sample was also calculated to compare the occurrence of nonsynonymous iSNVs among the three viruses. To calculate this proportion, the counts for nonsynonymous iSNVs was divided by the total counts of all iSNVs in a given sample. Similar to mean nonsynonymous iSNV frequency, virus genomes in OP swabs from turkeys infected with HPAIV had a lower proportion of nonsynonymous iSNVs than LPAIV-1 and LPAIV-2 (Figure 3C, Table 3). No differences were observed between LPAIV-1 and LPAIV-2 from either infected turkeys or from any groups for chickens. It thus appears that H7N3 LPAIVs had a greater fraction of nonsynonymous iSNVs, and at the same time, these iSNVs were found to have higher frequencies than that of the H7N3 HPAIV. This could indicate that a greater level of positive selective pressure was exerted to the H7N3 LPAIVs during within-host replication compared to their HPAIV counterpart.

#### 3.5.2. The LPAIV Genomes from Swabs Have Higher Genetic Diversity than the HPAIV Genomes

**Figure 4:**
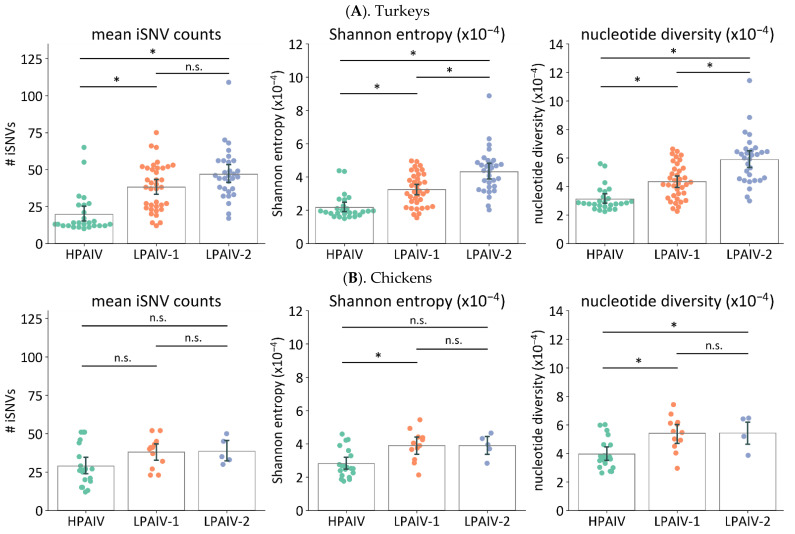
Comparison of iSNV diversity among the three H7N3 viruses in swab samples. Virus genomes from each swab sample from directly inoculated turkeys (**A**) and chickens (**B**) were sequenced and iSNVs identified. The iSNV diversity in each sample was examined by three measurements: mean iSNV count per sample, Shannon entropy, and nucleotide diversity. Asterisks (*) indicate *p* < 0.05 for indicated groups using Kruskal–Wallis and Dunn’s post hoc test for multiple comparisons. n.s. = not significant with *p* ≥ 0.05.

In the turkey samples, the mean number of iSNVs per sample were significantly lower in HPAIV than in LPAIV-1 or LPAIV-2, whereas no differences were observed between LPAIV-1 and LPAIV-2 (Figure 4A, Table 3). For Shannon entropy and nucleotide diversity, samples from infected turkeys had different levels of diversity in ascending order of value: HPAIV, LPAIV-1, and LPAIV-2 (Figure 4A, Table 3). For the chicken samples, virus genomes from HPAIV samples also had significantly lower Shannon entropy compared to that of LPAIV-1 and significantly lower nucleotide diversity compared to that of LPAIV-1 or LPAIV-2 (Figure 4B, Table 3). No differences were observed with diversity metrics between samples from chickens infected with LPAIV-1 and LPAIV-2 and with mean iSNVs per sample for all three viruses. Thus, the overall trend observed in swab samples was that virus genomes from birds infected with HPAIV had the least iSNV diversity, whereas those that came from birds infected with LPAIV-1 or LPAIV-2 had the higher iSNV diversity. Additionally, virus genomes from LPAIV-2-infected turkeys exhibited higher diversity compared to that from the LPAIV-1-infected turkeys.

### 3.6. Sample Type Can also Impact Nonsynonymous Changes and iSNV Diversity in Virus Genomes from Directly Inoculated Birds

#### 3.6.1. Frequencies and Proportion of Nonsynonymous Changes Are Higher in Tissue Samples than Swabs from Turkeys but Not Chickens

**Figure 5:**
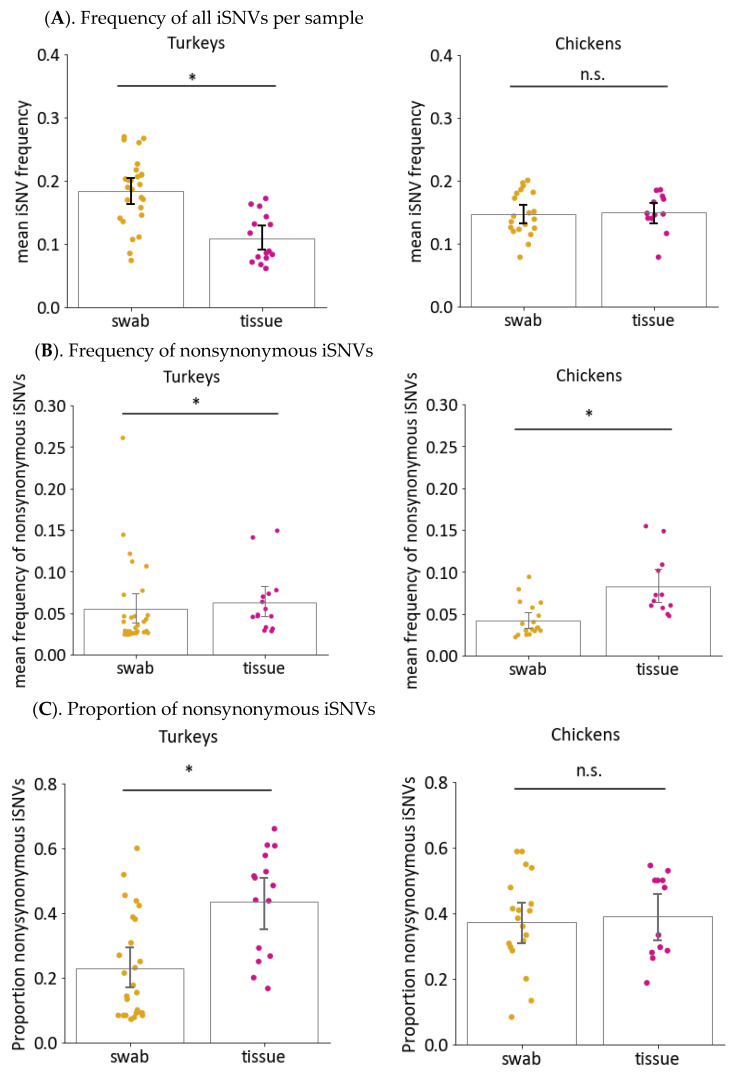
Frequency and proportion of nonsynonymous changes in swabs and tissues of HPAIV-infected turkeys and chickens. For each iSNV, the determination was made by whether an iSNV resulted in an amino acid change in at least one viral protein. The mean frequency of all iSNVs (**A**), and mean frequency (**B**) and proportion of nonsynonymous changes (**C**) in OP swabs or tissues samples from HPAIV-infected birds were then calculated. Asterisks (*) indicate *p* < 0.05 using Mann–Whitney test. n.s. = not significant with *p* ≥ 0.05.

It has been previously shown that there is heterogeneity in virus populations found in individual host tissues and that virus populations in different sites of replication are distinct, which can potentially have an impact on virus transmission and pathogenesis [49,50,51,52]. Only a small number of tissue samples were available for analyses, and thus, virus genome sequences from the five tissues, namely brain, heart, lung, muscle, and spleen, were considered together and compared to OP swabs. To further compare iSNVs in swab and tissue samples from HPAIV-infected birds, the mean frequency of all iSNVs and nonsynonymous iSNVs were also determined. The frequency of all iSNVs was significantly lower in tissues compared to swabs for turkeys, whereas no differences were observed for chickens (Figure 5A, Table 3). In contrast, a different trend was observed for nonsynonymous iSNVs. For both turkey and chicken samples, significantly higher frequencies of nonsynonymous iSNVs were observed in tissues compared to swabs (Figure 5B, Table 3). However, the proportion of nonsynonymous to synonymous changes between tissue and swab viruses were significantly different only in turkeys (Figure 5C, Table 3). The higher proportion and frequency of nonsynonymous virus changes in tissues samples of HPAIV-infected birds suggests that systemic HPAIV replication could potentially lead to faster virus adaptation through the accumulation of more amino acid changes.

#### 3.6.2. Viruses in Tissues Have Higher Genetic Diversity than Viruses in Swabs from Turkeys Infected with HPAIV

**Figure 6:**
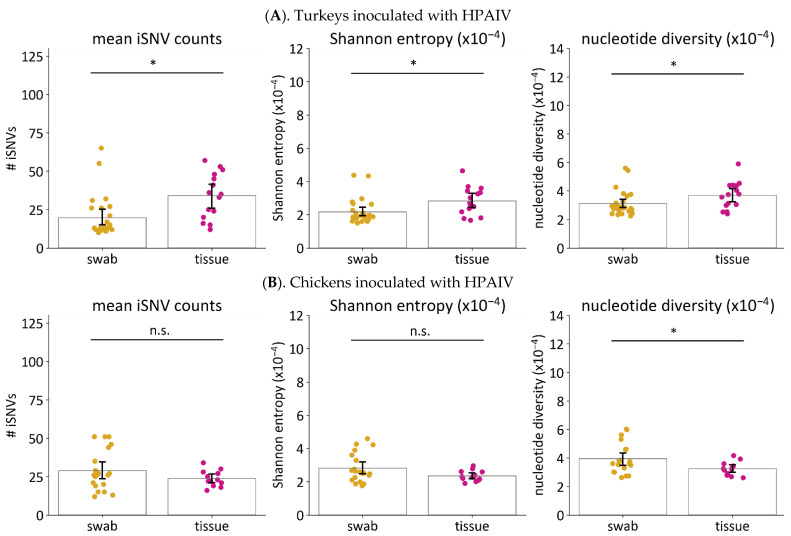
Comparison of iSNV diversity of H7N3 HPAIVs in tissues and swabs. The measures of iSNV diversity were calculated for OP swabs and tissue samples from directly inoculated turkeys (**A**) and chickens (**B**). The measures of iSNV diversity used are the mean iSNV count, Shannon entropy, and nucleotide diversity. Asterisks (*) indicate *p* < 0.05 using the Mann–Whitney test. n.s. = not significant with *p* ≥ 0.05.

In addition to OP swabs, we also calculated the iSNV diversity in viral genomes sequenced from brain, heart, lung, muscle, and spleen tissues from HPAIV-infected turkeys and chickens. No significant differences in iSNV diversity were observed among the five tissue types (*p* ≥ 0.05 using Kruskal–Wallis test). Because there was a small number of samples from each tissue type, we compared iSNV diversities between OP swabs and all five tissue samples considered together (Figure 6, Table 3). OP swabs and tissue samples from HPAIV-infected turkeys were found to have significant differences in all diversity metrics calculated. Specifically, we observed that the diversity metrics (mean iSNV counts, Shannon entropy, and nucleotide diversity) were higher in tissue samples compared to OP swabs. In contrast, for chickens, no significant differences in mean iSNV count and Shannon entropy were found. The only significant difference observed was with nucleotide diversity which was higher in OP swabs compared to tissue samples.

### 3.7. Some iSNVs in Experimental Samples Were Found in Single Nucleotide Changes in the Outbreak Sequences

**Figure 7:**
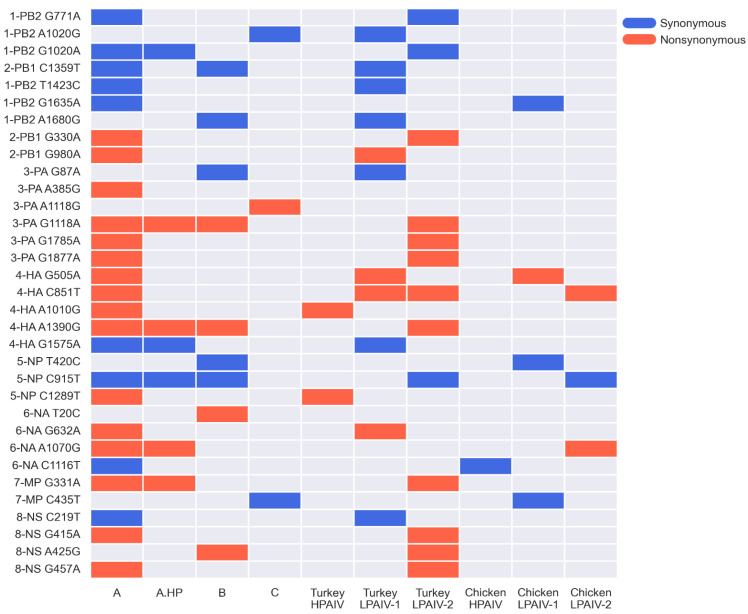
Matrix of common SNVs found in iSNVs, and pairwise comparisons of all H7N3 viruses sequenced from the 2020 US outbreak. The iSNVs identified in samples from experimentally infected turkeys and chickens were compared against the single nucleotide changes that were found in all possible pairwise comparisons among all twenty-nine virus isolates during the 2020 US H7N3 outbreak [30]. On the left of the matrix, the nucleotide position of the iSNV in the corresponding segment is flanked by two letters that show the nucleotide substitution from left to right. Squares in red or blue indicate the presence of the corresponding iSNV to the left of the matrix. Red squares represent nonsynonymous iSNVs, whereas blue squares represent synonymous iSNVs. Outbreak sequences were also categorized according to the phylogenetic clusters A, A. HP, B, and C identified previously [30].

To determine whether within-host iSNVs were also found in the outbreak sequences, all possible pairwise sequence comparisons were performed using full genome sequences from all 29 outbreak viruses previously reported [30]. The single nucleotide changes between all possible pairwise sequence comparisons were then noted. Excluding the insertion of the multibasic cleavage site (MBCS) in the HA gene and the 66-nucleotide deletion in the NA gene, a total of 257 unique single nucleotide differences were observed between all pairwise comparisons of the 29 outbreak sequences. Of these changes, 33 correspond to the iSNVs identified in genomes sequenced from experimental OP swabs and tissue samples (Figure 7). These 33 iSNVs were distributed across the eight gene segments. Nineteen of these changes were found to be nonsynonymous changes. Furthermore, most of these changes were found in the largest cluster of outbreak viruses called Cluster A, and in LPAIV-1 or LPAIV-2 genome sequences. Only 8 out of the 33 common changes were observed in the chickens experimentally infected with LPAIV-1, LPAIV-2, or HPAIV. To our knowledge, the 33 common changes have not been previously implicated in any change in virus phenotype.

## 4. Discussion

Avian influenza viruses (AIVs) are some of the most important viruses threatening animal and human health due to its vast genetic diversity and relatively wide host species range [53,54,55]. Similar to other RNA viruses, AIVs have a high mutation rate, short generation times, and are present in large populations, all contributing to the rapid generation of diversity within hosts upon infection, which is ultimately the source of genetic diversity in longer time scales and geographic space [4,6,56]. There has been increasing appreciation of the fact that consensus sequences that are reported in public repositories only represent the most abundant viruses in a sample and overlook low-frequency variants that can potentially expand under the appropriate circumstances. Additionally, it has been shown that traditional Sanger sequencing cannot resolve variations of less than 20% in clinical samples from patients with human immunodeficiency virus (HIV) [22]. In contrast, short-read deep sequencing can routinely resolve virus variants at ≥2% frequencies [4,21,44,46,47,57]. Indeed, the advent of accessible, cost-efficient high throughput sequencing has allowed investigators to examine viruses as populations composed of closely related variants in a host [4,57].

This study examined within-host virus populations of three H7N3 AIVs in two agriculturally important poultry species—turkeys and chickens. Total RNA obtained from oropharyngeal (OP) swabs and tissue samples from experimentally infected turkeys and chickens was subjected to AIV full-genome amplification, deep sequencing, genome assembly, and identification of intra-host single nucleotide variants (iSNV). Consistent with prior studies on avian and human seasonal influenza viruses [6,58,59,60], most of the iSNVs were present at low frequencies of less than 10% (Figure 1 and Supplementary Figures S1 and S2). Additionally, comparison of iSNVs within hosts and those viruses isolated from the outbreak showed that only a small number of iSNVs correspond to sequences of outbreak viruses (Figure 7), similar to what was found in another study in which iSNVs were examined in H5N1-infected humans and ducks in Cambodia [6]. The existence of many low frequency iSNVs across different individual hosts most likely reflects the high mutation rates of AIVs [61,62]. Furthermore, the observation that there are few iSNV types shared between hosts (Supplementary Figure S3) and between contact-transmission groups (Figure 2), suggest that many of these low frequency iSNVs were generated de novo during replication in an individual host.

In a longer view of virus evolution, the existence of low-frequency iSNVs can allow the virus population to be collectively resilient to changing selective pressures because these low frequency iSNVs can exponentially expand to adapt to prevailing circumstances. The high mutation rate of AIVs within hosts can thus be an important strategy for virus adaptation. Indeed, it has been estimated that influenza A viruses incur on average 2–3 mutations per replicated genome within a cell, and that the mutation rate during replication in a cell is substantially higher than what is observed in longer time scales such as those during an epidemic [63]. Interventions to increase the mutation rates of influenza A viruses either through antiviral drug treatment or amino acid changes in polymerase proteins have led to error catastrophe, further demonstrating that influenza A viruses operate close to the theoretical maximum mutation rate [64,65,66]. The preponderance of low-frequency iSNVs within a host does not appear to be unique to influenza viruses and could be a strategy for diversification and adaptation in other RNA viruses [67,68]. It has been shown for instance that the within-host mutation rate of yellow fever virus is about 2.25 times higher than that observed during an epidemic, and 59 times higher compared to longer evolutionary timescales [67,68]. Furthermore, alteration of viral RNA polymerases to increase fidelity from several viral species, including influenza A, have been shown to have an attenuating effect, presumably because of the lack of viral diversity in the infecting populations [65,69,70,71].

In samples from both turkeys and chickens, there were generally more iSNVs that resulted in an amino acid change in at least one protein (nonsynonymous) than those that did not (synonymous), especially for LPAIV-1 and LPAIV-2 (Figure 1 and Figure 3). The preponderance of nonsynonymous iSNVs suggests that the H7N3 had recently spilled over from the wild bird reservoir to turkeys, and virus adaptation to a new host was underway during the outbreak. In this study, we did not measure the iSNVs in a wild bird host such as mallards. Nonetheless, it has been shown that the overall substitution rate and dN/dS ratio of AIVs is lower in wild birds than in domestic poultry species and is likely a reflection of its AIV reservoir status [72,73]. Furthermore, experimental infections of a turkey-derived H9N2 virus has also shown that the highest number of nonsynonymous changes occurred in samples from infected ducks, most likely reflecting shifts in host species [11]. Similarly, experimental passage of avian, canine, or seasonal human influenza A viruses in embryonating eggs or immortalized cell lines have shown the preponderance of nonsynonymous changes that has been attributed to shifts in viral replication from its natural host to an experimental culture system [7,74,75]. This increase in nonsynonymous changes coinciding with a change in host has been further documented for a variety of viruses including severe acute respiratory syndrome coronavirus-2 (SARS-CoV-2) [20], Newcastle disease virus [13], bovine viral diarrhea virus [76]. For example, in patients infected with human immunodeficiency virus (HIV), it has been shown that high sequence diversity and nonsynonymous changes occur early during acute infection that eventually plateaus during the chronic phase [14].

Enumeration of unique iSNVs for viruses sequenced from H7N3 infected birds showed that only a few iSNVs are shared between turkeys and chickens (Supplementary Figure S3), also highlighting the preponderance of de novo mutations. The lack of overlap of iSNVs between the two host species may also suggest that adaptation to turkeys may follow a different path when the same virus is allowed to adapt to chickens. Nonetheless, we observed five iSNVs that were ≥ 50% in frequency and were found in at least one turkey and one chicken sample (Table 2). These iSNVs are potentially changes that allow for further virus adaptation to gallinaceous avian species. Further studies will be needed to determine if this is the case.

We did not observe any significant differences in measures of iSNVs diversity when comparing dose, route of exposure, and time after exposure for each virus. The lack of inter-relationship of iSNV diversity and route of exposure, dose, or time after infection may suggest that intrinsic virus–host interactions play a larger role in determining within-host virus diversity of these H7N3 viruses in turkeys and chickens. Potentially, within-host diversity can be modulated by differences in host cell requirements for viral replication or innate antiviral responses and could also explain the differences in susceptibility between turkeys and chickens [3,77]. Further investigation will be needed to determine if such host factors play a role.

We also compared the iSNVs that are shared between directly inoculated and contact-exposed turkeys and found that there is also little overlap between the two groups (Figure 2). This may indicate that some bottleneck exists during transmission, potentially due to the small number of viral particles that are transmitted to contacts [78]. Other groups working with influenza A viruses and other virus species have also observed similar phenomena and have shown that the mode of transmission can impact viral diversity in recipient hosts [9,12,20,57,59,79,80,81]. For instance, it has been shown that influenza A viruses transmitted through aerosol place a more stringent bottleneck than those transmitted through direct contact, presumably because fewer viral particles are transmitted through aerosol than direct contact [9]. Additionally, the role of multiple rounds of transmission on viral diversity should be further explored as it could have compounding effects on virus population bottlenecks.

Viral diversity can be described through the number of iSNVs in each sample, or a metric such as Shannon entropy and nucleotide diversity [4,7,11,13,44,81,82]. In this study, we observed that LPAIV-1 and LPAIV-2 had higher iSNV diversity than the HPAIV (Figure 4). Similar trends were also observed for the frequency and proportion of nonsynonymous iSNVs (Figure 3). Because the three viruses were closely related genetically, these observations suggest that the change in pathogenicity type from LPAIV to HPAIV has some impact on within-host diversity. This can potentially be due to the relatively rapid disease progression of HPAIVs compared to its LPAIV counterparts, which may provide fewer replication cycles and a smaller timeframe for the virus to accumulate variants within the population, and for the host defenses to intervene during virus replication. Because the inoculum of HPAIV was serially passaged in 14-day-old embryos in order to isolate HPAIV from a mixture of HPAI and LPAIVs [35], we also considered another possibility that its passage history may have led to less diverse pools of viruses. However, the measurement of iSNV diversity in the three virus inocula appears to be comparable to each other (Table 3).

Deletions in the NA stalk region have been described to emerge during outbreaks [30,83,84] and experimental passage in poultry [56,85]. It is a common mutation found across different AIV subtypes [86] and has been proposed as an adaptation strategy for replication in gallinaceous species [87]. In our previous pathogenesis study, the LPAIV-2 virus with the NA-stalk deletion required a slightly lower mean bird infectious dose in both turkeys and chickens [35]. In this study, we found that LPAIV-2 had higher iSNV diversity than LPAIV-1 in turkeys (Figure 4), consistent with the role of the NA stalk-deletion in increasing adaptation to turkey hosts. No differences in iSNV diversity were observed in chickens between LPAIV-1 and LPAIV-2, suggesting that the impact of NA-stalk deletion may be specific to turkeys.

Differences in viral diversity between different sites of replication have been documented for influenza viruses [49,50] as well as for SARS-CoV-2 [51] and coxsackievirus B3 [52]. These studies have shown that viral diversity was restricted in a tissue-specific manner. The existence of distinct virus populations in different sites of replication can have potential impact on the viruses that are transmitted or those viruses that cause certain disease [50]. Consistent with the other studies, viral genomes sequenced in swabs and tissues from HPAIV-infected turkeys were found to have different intra-host iSNV diversities (Figure 6). Moreover, the frequency and proportion of nonsynonymous iSNVs was greater in tissues compared to swabs, specifically for samples from infected turkeys (Figure 5). The higher iSNV diversity and nonsynonymous iSNVs in tissues may reflect differences in selective pressures in different sites of replication. Thus, the H7N3 HPAIV appears to encounter less stringent selective pressures during systemic replication in turkeys. In HPAIV-infected chickens, the lack of differences in viral diversity between swabs and tissues suggests that the selective pressures between these replication sites are comparable.

## 5. Conclusions

In this study, we have shown that the viral diversity within hosts can vary even among closely related avian influenza viruses. Specifically, the presence of a multibasic cleavage site in the HA, which expands the tissue tropism of the HPAIV, was associated with overall lower within-host viral diversity compared to its LPAIV counterparts. Studies using other sets of influenza viruses will need to be conducted to determine if this is a general association with HPAIV or if this is specific to the set of viruses tested here. Our results also highlight the abundance of low-frequency nonsynonymous iSNVs observed within hosts, consistent with the high mutation rate of influenza viruses. These low-frequency iSNVs could be sources of genetic diversity that could overcome selective pressures and result in the emergence of new virus variants. It would be valuable to further investigate scales of time and space that bridge within-host changes and those used for phylodynamic analyses to better understand influenza virus diversification and virus evolution at large.

## Figures and Tables

**Table 1:** Samples sequenced for this study.

Virus	Species	Exposure	Sample Type	# Samples ^a^	# Birds ^b^
LPAIV-1	Turkey	inoculated	OP	37/56	10/10
	Chicken		OP	12/33	6/9
LPAIV-2	Turkey		OP	30/94	13/15
	Chicken		OP	5/30	5/10
HPAIV	Turkey		OP	26/33	15/15
			tissues	15/15	3/3
	Chicken		OP	20/23	13/14
			tissues	12/15	3/3
LPAIV-1	Turkey	contact	OP	13/17	2/2
LPAIV-2			OP	6/17	2/2
HPAIV			OP	4/12	2/2
			Total	180/345	74/85

^a^ Fraction of samples from which viral genomes were successfully sequenced to the total number of samples positive for viral RNA by qRT-PCR. ^b^ Fraction of birds from which the samples were successfully sequenced to the total number of infected birds.

**Table 2:** The iSNVs at frequency greater than or equal to 50% and are found in at least one sample from turkeys and chickens.

				Number of Samples ^a^				
				Turkeys			Chickens	
				Inoculated		Contact	Inoculated	
Virus	Segment	SNV	Amino Acid Change	OP	Tissue	OP	OP	Tissue
LPAIV-1	NP	T507C	synonymous	2/37	n.a.	1/13	3/12	n.a.
LPAIV-2	PB1	G183A	PB1-F2 G22E	4/30	n.a.	0/6	1/5	n.a.
LPAIV-2	NS	G485A	E153K	19/30	n.a.	6/6	2/5	n.a.
HPAIV	PA	T1489A	C489S	0/26	3/15	0/4	0/20	2/12
HPAIV	NS	T294C	L90P	6/26	5/15	2/4	3/20	0/12

^a^ Number of samples with iSNV frequency ≥ 50%/total number of samples. n.a. = sample not available.

**Table 3:** Diversity measures in inocula and samples. The mean values for viral genomes from experimental samples were calculated for the indicated diversity measures. n.a. = not applicable.

Sample Type	Virus	Species	iSNV Frequency	Nonsynonymous iSNV Frequency	Proportion of Nonsynonymous iSNVs	SNV Count	Shannon Entropy (×10^−4^)	Nucleotide Diversity (×10^−4^)
swab	HPAIV	Chicken	0.15	0.045	0.374	28.95	2.81	3.95
		Turkey	0.19	0.050	0.231	19.23	2.16	3.10
	LPAIV-1	Chicken	0.15	0.084	0.473	38.00	3.90	5.41
		Turkey	0.14	0.080	0.477	37.86	3.28	4.40
	LPAIV-2	Chicken	0.16	0.116	0.521	38.60	3.89	5.43
		Turkey	0.16	0.099	0.511	47.53	4.29	5.83
tissue	HPAIV	Chicken	0.15	0.085	0.392	23.75	2.35	3.25
		Turkey	0.11	0.056	0.436	34.07	2.83	3.69
inocula	HPAIV	n.a.	0.11	0.065	0.281	48.00	4.42	5.93
	LPAIV-1	n.a.	0.11	0.071	0.255	41.00	3.59	4.74
	LPAIV-2	n.a.	0.10	0.080	0.307	50.00	4.26	5.00

## Data Availability

Inoculum sequences have the following GenBank accession numbers: A/turkey/South Carolina/20-008394-1/2020 (LPAIV-1) (GenBank accession number MT444368-MT444375), A/turkey/North Carolina/20-008425-1/2020 (LPAIV-2) (GenBank accession number MT444287-MT444294), and A/turkey/South Carolina/20-010561-006/2020 (H7N3) (HPAIV) (GenBank accession number OL782155-OL782162). Twenty-nine whole genome sequences were obtained from the 2020 H7N3 outbreak in turkeys [30], which have GenBank accession numbers MT444183–350 and MT444352–415. Raw sequence reads from experimental samples were deposited in the Sequence Read Archive with BioProject number PRJNA792828.

## Supplementary Materials

The following supporting information can be downloaded at: https://www.mdpi.com/article/10.3390/v14030554/s1, Figure S1: Distribution of iSNVs across the genome; Figure S2. Mean iSNV frequency by virus gene segment; Figure S3. Common iSNVs found in turkeys and chickens.
