# Supplementary material for: Low Pathogenicity H7N3 Avian Influenza Viruses Have Higher Within-Host Genetic Diversity Than a Closely Related High Pathogenicity H7N3 Virus in Infected Turkeys and Chickens
Source: Viruses. 2022 Mar 8;14(3):554. doi: 10.3390/v14030554 (PMC8951284; doi:10.3390/v14030554)

Figure S1. Distribution of iSNVs across the genome. Genome location of all iSNVs identified from turkeys (A-D) and chickens (E-H) experimentally infected with one of three H7N3 AIVs. All iSNVs from every sample in the indicated category were pooled and plotted along its position on the concatenated genome of the indicated H7N3 virus.

(A)

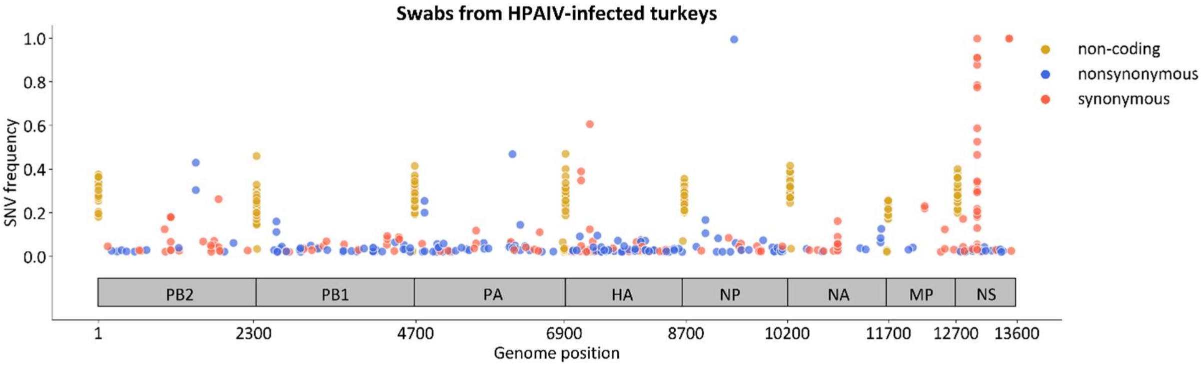

(B)

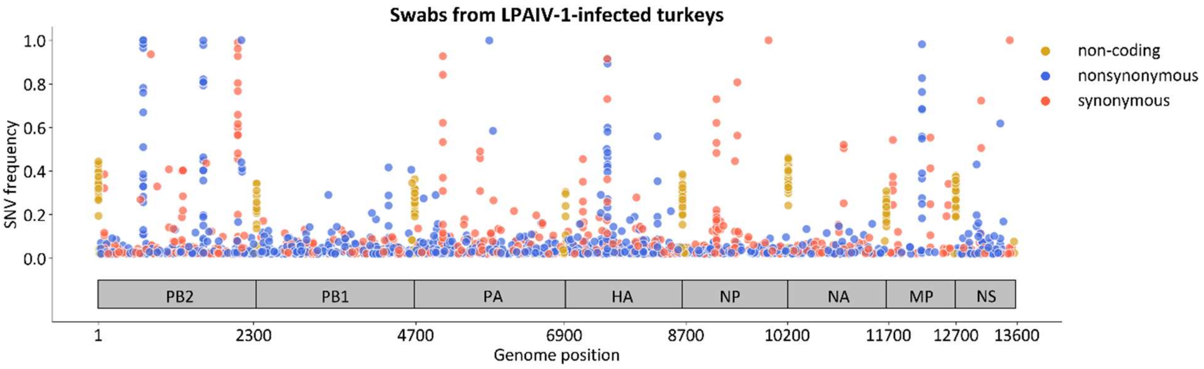

(C)

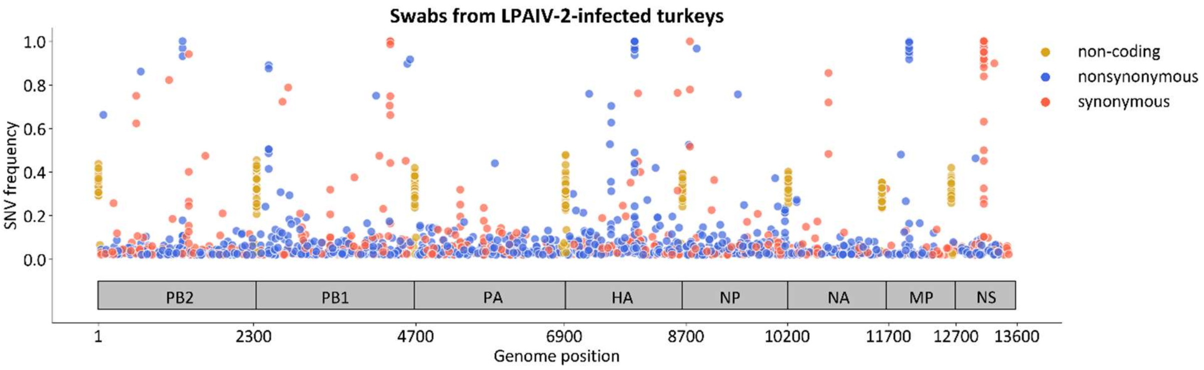

(D)

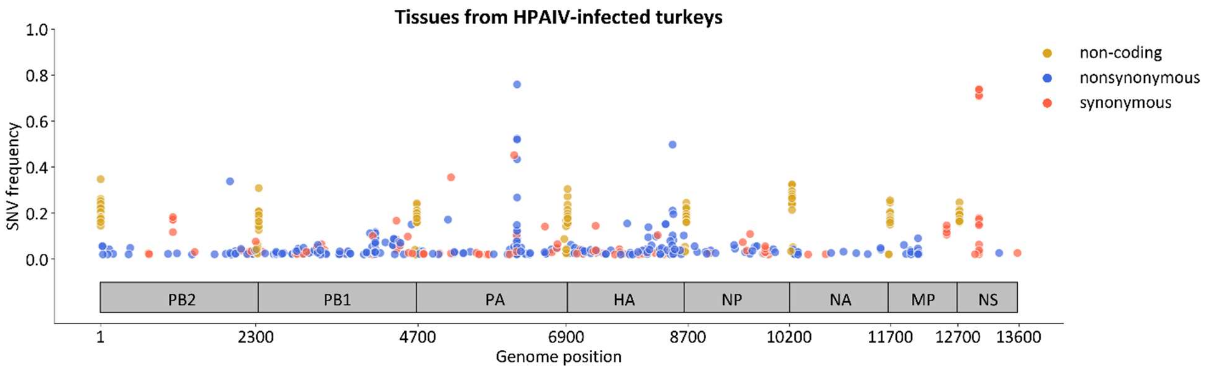

Tissues from HPAIV-infected turkeys
SNV frequency
1.0
0.8
0.6
0.4
0.2
0.0
non-coding
nonsynonymous
synonymous
PB2
PB1
PA
HA
NP
NA
MP
NS
1
2300
4700
6900
8700
10200
11700
12700
13600
Genome position

(E)

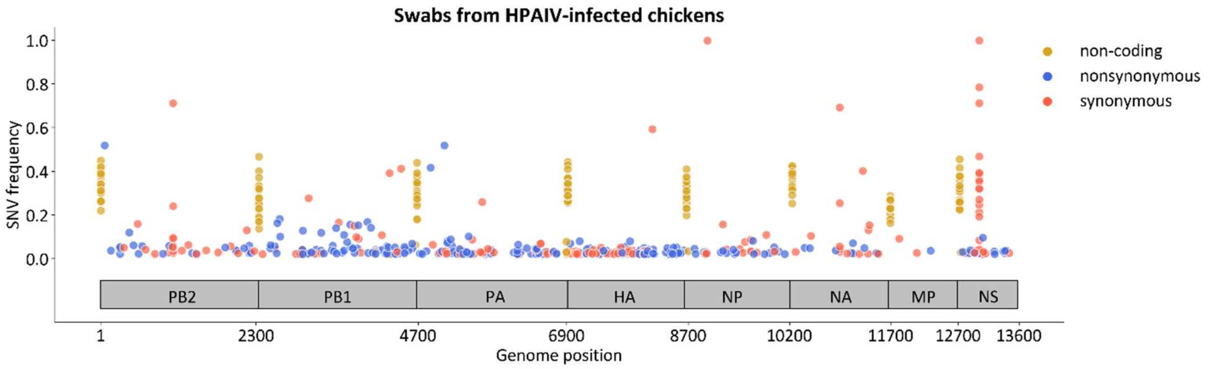

Swabs from HPAIV-infected chickens
SNV frequency
1.0
0.8
0.6
0.4
0.2
0.0
non-coding
nonsynonymous
synonymous
PB2
PB1
PA
HA
NP
NA
MP
NS
1
2300
4700
6900
8700
10200
11700
12700
13600
Genome position

(F)

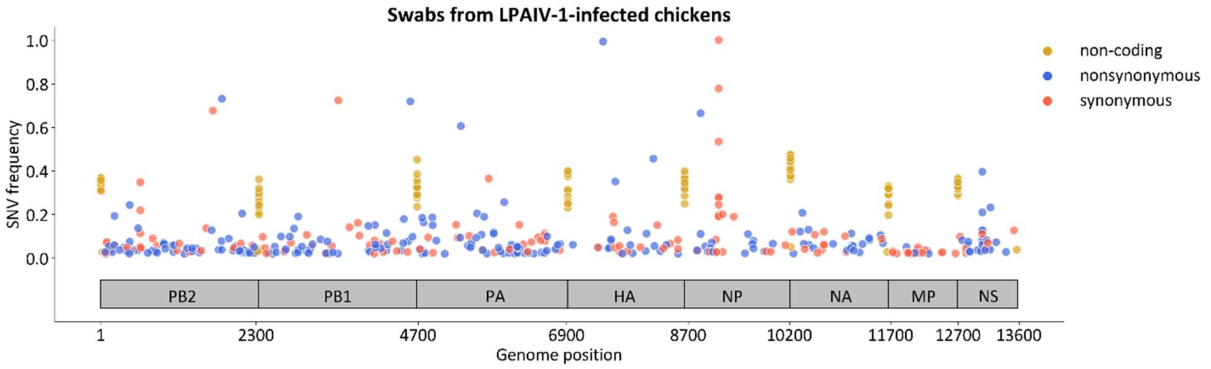

Swabs from LPAIV-1-infected chickens
SNV frequency
1.0
0.8
0.6
0.4
0.2
0.0
non-coding
nonsynonymous
synonymous
PB2
PB1
PA
HA
NP
NA
MP
NS
1
2300
4700
6900
8700
10200
11700
12700
13600
Genome position

(G)

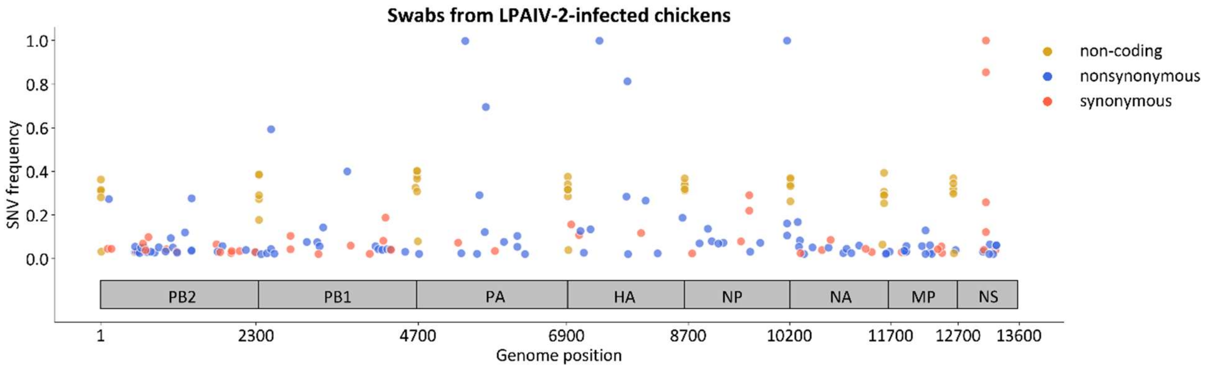

Swabs from LPAIV-2-infected chickens
1.0
0.8
0.6
0.4
0.2
0.0
SNV frequency
non-coding
nonsynonymous
synonymous
PB2
PB1
PA
HA
NP
NA
MP
NS
1
2300
4700
6900
8700
10200
11700
12700
13600
Genome position

(H)

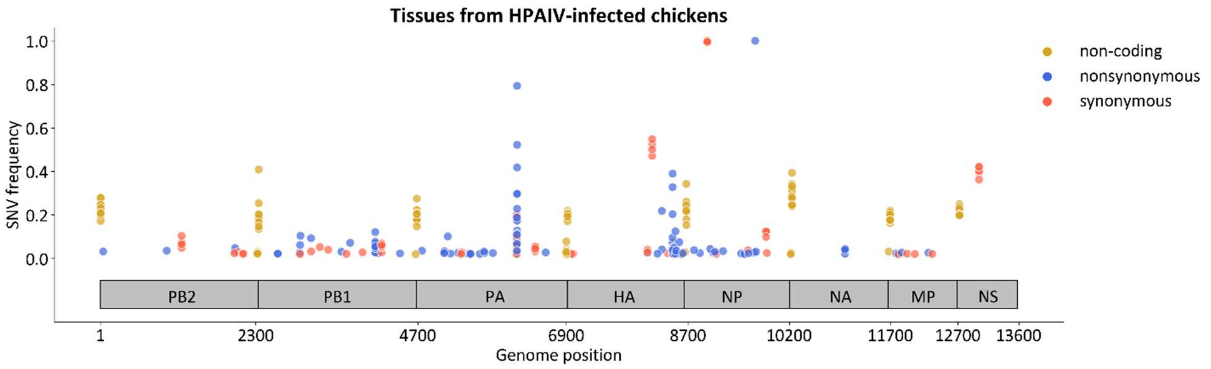

Tissues from HPAIV-infected chickens
1.0
0.8
0.6
0.4
0.2
0.0
SNV frequency
non-coding
nonsynonymous
synonymous
PB2
PB1
PA
HA
NP
NA
MP
NS
1
2300
4700
6900
8700
10200
11700
12700
13600
Genome position

Figure S2. Mean iSNV frequency by virus gene segment. The mean frequency of iSNVs was calculated per segment for each sample. The dotted line indicates a frequency of 0.5 or 50%.

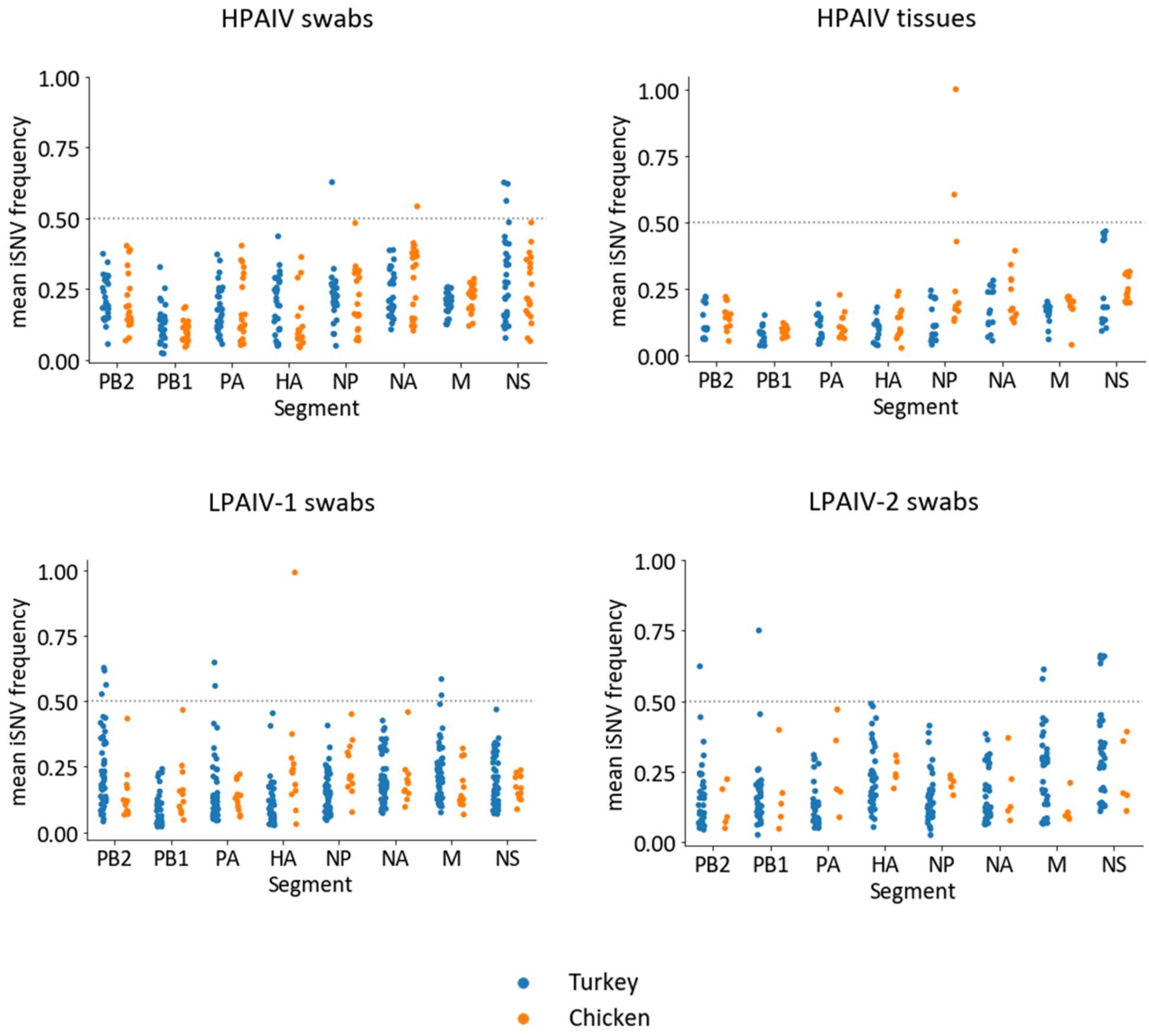

Figure S3. Common iSNVs types found in turkeys and chickens. The iSNVs from each sample were enumerated and pooled from samples in the same host species. The sets of unique iSNVs were then compared between samples from turkeys and chickens.

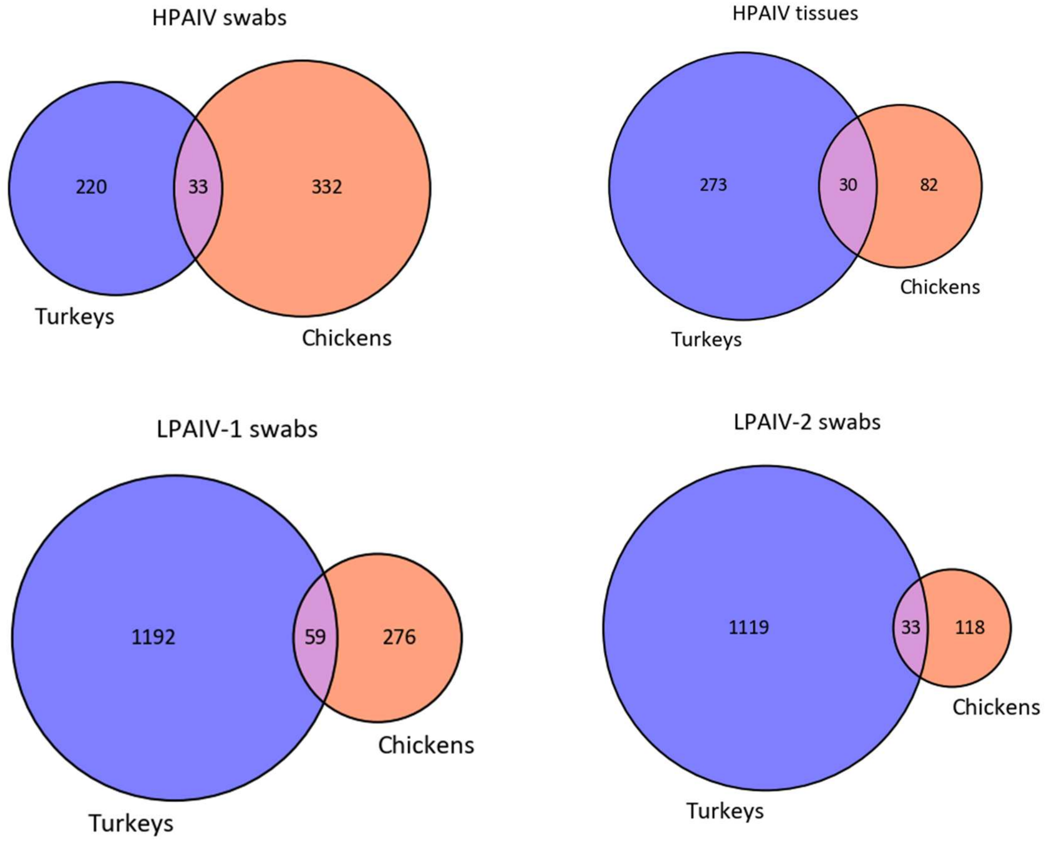

Supplement: Supplementary file 1 [file viruses-14-00554-s001.zip › viruses-1611535-supplementary.pdf]
